# Development and characterization of EST‐SSR markers for *Camellia reticulata*

**DOI:** 10.1002/aps3.11348

**Published:** 2020-05-22

**Authors:** Yan Tong, Li‐Zhi Gao

**Affiliations:** ^1^ Yunnan Key Laboratory for Wild Plant Resources Kunming Institute of Botany Chinese Academy of Sciences 132 Lanhei Road Kunming 650201 Yunnan China; ^2^ Germplasm Bank of Wild Species Kunming Institute of Botany Chinese Academy of Sciences 132 Lanhei Road Kunming 650201 Yunnan China; ^3^ Institution of Genomics and Bioinformatics South China Agricultural University 483 Wushan Road Guangzhou 510642 Guangdong China

**Keywords:** *Camellia reticulata*, expressed sequence tag–simple sequence repeat (EST‐SSR) marker, polymorphism, Theaceae

## Abstract

**Premise:**

*Camellia reticulata*, which is native to southwestern China, is an economically important plant belonging to the family Theaceae. We developed expressed sequence tag–simple sequence repeat (EST‐SSR) markers for *C. reticulata*, which can be used to investigate its genetic diversity, population structure, and evolutionary history.

**Methods and Results:**

We detected 4780 SSRs in *C. reticulata* from *Camellia* RNA‐Seq data deposited in the National Center for Biotechnology Information's expressed sequence tags database (dbEST). Primer pairs for 70 SSR loci were designed and used for PCR amplification using 90 individuals from four populations of *C. reticulata*. Of these loci, 50 microsatellite markers were successfully identified, including 11 polymorphic markers. The allele number per locus ranged from two to seven (mean = 4.182), and the levels of observed and expected heterozygosity ranged from 0.044 to 0.567 and from 0.166 to 0.642, respectively. Eleven primer pairs amplified PCR products in three other species of *Camellia* (*C. saluenensis*,* C. pitardii*, and *C. yunnanensis*).

**Conclusions:**

The set of microsatellite markers developed here can be used to study the genetic variation and population structure of *C. reticulata* and related species and thereby help to develop conservation strategies for this species.

*Camellia reticulata* Lindl. (Theaceae) is an economically important ornamental flowering shrub or small tree that grows in Yunnan Province, southwestern Sichuan Province, and western Guizhou Province of China (Ming et al., 2000). As an ornamental plant, *C. reticulata* has over 1000 years of history of cultivation, and many outstanding cultivars have been selected or bred from wild *C. reticulata* for many centuries in China (Xia et al., 1994; Gu, 1997). *Camellia reticulata* is notable for its large flowers, brilliant colors, numerous cultivars, and long flowering duration (Xia et al., 1994; Ming et al., 2000). Furthermore, it is valued not only as a flowering ornamental but also as a source of oils. Its seeds have a high content of oil that is rich in unsaturated fatty acids, oleic acid, vitamin E, and other physiologically active substances, making it a valuable edible oil source for daily consumption (Liu and Ma, 2010). *Camellia reticulata* is a perennial, outcrossing, heterogenous polypoid species (2*n* = 30) (Kondo et al., 1986). Its natural populations across southwestern China have different ploidy levels (i.e., 2*n* = 2*x*, 4*x*, 6*x*;* x* = 15), which could have resulted from natural hybridization and polyploidization (Gu, 1997). Fluorescence in situ hybridization and genomic in situ hybridization for *C. reticulata* indicate that *C. japonica* L., *C. saluenensis* Stapf ex Bean, *C. pitardii* Cohen‐Stuart, and *C. yunnanensis* (Pit. ex Diels) Cohen‐Stuart might have contributed to the origin and evolution of the *C. reticulata* polyploid complex (Gu and Xiao, 2003; Liu and Gu, 2011).

In recent years, there has been a decline in the number and size of natural populations of *C. reticulata* because of overharvesting and habitat destruction. This alarming situation necessitates an in‐depth understanding of the current status of the genetic diversity of the species. Studies have been conducted on the genetic diversity of *C. reticulata* via inter‐simple sequence repeat markers, chloroplast microsatellites, and amplified fragment length polymorphisms (Wang and Ruan, 2012; Tong et al., 2013; Yao et al., 2016; Xin et al., 2017). Yao et al. (2016) designed 20 expressed sequence tag–simple sequence repeat (EST‐SSR) primer pairs based on the transcriptome of diploid *C. reticulata*. Of these, 18 were successfully amplified, detecting seven polymorphic loci in 24 *C. reticulata* individuals. We further tested these 20 markers in four natural populations, showing four loci with polymorphisms. These are not sufficient for inclusive studies on *C. reticulata*. Therefore, in this study, we aimed to develop new microsatellite markers that will help to investigate the reproductive characteristics of *C. reticulata*, evaluate its evolutionary potential, and develop effective strategies for the conservation, development, and utilization of wild natural populations. In addition, we tested the cross‐species transferability of these markers in three other species of *Camellia*:* C. saluenensis*,* C. pitardii*, and *C. yunnanensis*, which are thought to be involved in the polyploidy of *C. reticulata*.

## METHODS AND RESULTS

Fresh healthy leaves collected from 90 individuals of *C. reticulata* sampled from four wild populations from Yunnan Province, China, were freeze‐dried or silica‐dried. Forty samples from three populations of *C. saluenensis*,* C. pitardii*, and *C. yunnanensis* were also collected to test the cross‐amplification of the markers. Voucher specimens were deposited at the Kunming Institute of Botany, Chinese Academy of Sciences (KUN) (Appendix 1). Genomic DNA was extracted from 20–30 mg of dried leaf tissue using a modified cetyltrimethylammonium bromide (CTAB) method (Doyle and Doyle, 1987).

We obtained 50,287 EST sequences from the National Center for Biotechnology Information (NCBI) expressed sequence tags database (dbEST) (accessed on June 2019) (Boguski et al., 1993). To obtain a nonredundant EST data set for SSR identification and primer design, vectors were removed from EST sequences using SeqTrim (Falgueras et al., 2010) and poly(A) tails were trimmed using est‐trimmer.pl. Clean EST sequences were then clustered and assembled into contigs and singletons using CAP3 (Huang and Madan, 1999), generating 17,989 unigenes consisting of 5099 contigs and 12,890 singletons. These unique sequences were further used to screen for the presence of microsatellites using the MISA Perl program (Thiel et al., 2003). The criteria for SSRs were set as sequences having at least 10, six, five, five, five, and five repeat units for mono‐, di‐, tri‐, tetra‐, penta‐, and hexanucleotide motifs, respectively. In total, 4780 SSRs were identified, with an average frequency of 1 SSR/1.57 kbp. Primers were designed using Primer Premier 5.0 software (PREMIER Biosoft International, Palo Alto, California, USA) with the following criteria: primer lengths of 16–22 bp, GC content of 40–65%, annealing temperature ranging from 40°C to 60°C, and a predicted PCR product size ranging from 100 to 300 bp.

We randomly selected 70 primer pairs and tested them for PCR amplification in 12 accessions of *C. reticulata* (three individuals in each population, Appendix 1) in an initial screening test. PCR amplification was performed in an 18‐μL reaction mixture containing 20–30 ng of genomic DNA, 9 μL of 2× Easy Taq PCR Super Mix (TransGen Biotech, Beijing, China), and 1 μL of each primer (10 μM), adding ddH_2_O to a final volume of 18 μL. Cycling consisted of 30 s of denaturation at 94°C, 30 s at the optimized annealing temperature (Table 1), and a 1‐min extension at 72°C for 32 cycles, followed by a final extension at 72°C for 5 min. The amplified products were separated on 8% polyacrylamide denaturing gels, and the bands were developed with silver staining with a 2‐kbp DNA Ladder Marker (Hangzhou Bioer Technology Co. Ltd., Hangzhou, China) as a reference. The ploidy level of the sampled populations was unknown, but multiple copy bands per locus due to polyploidy were not observed. Of the 70 primer pairs tested, 50 yielded clear and reproducible amplicons in *C. reticulata*; the others were unstable or gave no product. Eleven loci showed polymorphisms (Table 1), and 39 loci were monomorphic (Appendix 2). These 11 polymorphic primers were used in 90 individuals of *C. reticulata* (four populations) for the population genetic analyses using the same protocol as the initial test. The polymorphic SSR loci were analyzed with POPGENE 32 software (Yeh et al., 2000) and GenAlEx (Peakall and Smouse, 2006) for the number of alleles per locus, observed heterozygosity, and expected heterozygosity (Table 2). Hardy–Weinberg equilibrium by 1000 randomizations and linkage disequilibrium were estimated using POPGENE 32 software (Yeh et al., 2000).

**Table 1:** Characteristics of 11 polymorphic microsatellite loci developed in *Camellia reticulata*.^a^

Locus	Primer sequences (5′–3′)	Repeat motif	Expected allele size (bp)	*T*_a_ (°C)	Putative function (Organism)	GenBank accession no.
CSSR2	F: GGAATAGGCTCGGATGGT	(TTG)_7_	186	54	Hypothetical protein TEA_012945 (*Camellia sinensis* var. *sinensis*)	FS951581.1
	R: CTCTCTGCTTCTTCACAAATC					
CSSR5	F: GCTGTAGGCGAACATGAA	(GGTGCT)_6_	196	55	Glycine‐rich cell wall structural protein 1.8‐like (*Camellia sinensis*)	FS952802.1
	R: CACTTCCACTTCCATATCCA					
CSSR11	F: GCCCTAACTCTTCACTGTA	(AG)_18_	123	56	Growth‐regulating factor 4‐like isoform X2 (*Camellia sinensis*)	FS950234.1
	R: CTATGTCGGCTAGGTTCTT					
CSSR17	F: AGAGGAGAGGAGAGGAGAG	(CCTCCA)_7_	130	54	NONE	GH710908.1
	R: TTTGGAGAGCGACATTGC					
CSSR18	F: TCGCTGCTCTCATCTACT	(CTT)_9_	114	56	Hypothetical protein TEA_017838 (*Camellia sinensis* var. *sinensis*)	FS945416.1
	R: TCTACATGGACATGGACTTAG					
CSSR19	F: GCTCATGCCATGTCATCC	(CT)_12_	167	55	sm‐like protein LSM1B (*Camellia sinensis*)	GH710926.1
	R: TACCCTCATATCAACCTTGTG					
CSSR35	F: ATCGCAGACAACAAGAAGA	(TGA)_6_	105	55	Probable E3 probable E3 ubiquitin‐protein ligase XERICO (*Camellia sinensis*)	FS947941.1
	R: GGAGGAGATCGTGATGAAG					
CSSR36	F: AGGCTTAGGTGTAGATAGGT	(TC)_16_	117	54	Histone H1‐like protein (*Camellia sinensis*)	JK711494.1
	R: ACTCCAACTCTTCCACAAC					
CSSR38	F: GCTATTGACGCTAATGACC	(TGA)_6_	117	55	Protein PAT1 2 like (*Actinidia chinensis* var. *chinensis*)	FS949009.1
	R: CCAGAAATCATAACGCAACA					
CSSR45	F: GTATGACAGATACCATGAACC	(T)_21_	157	55	Hypothetical protein TEA_005759 (*Camellia sinensis* var. *sinensis*)	FF682781.1
	R: TGAAACCAAACCCACACT					
CSSR48	F: ATTACCACCACCACTATCAC	(TGG)_8_	143	55	ABA‐inducible protein PHV A1‐like (*Camellia sinensis*)	GH738605.1
	R: CCCAAAGAAAGACCAAAGAC					

Note

*T*_a_ = annealing temperature.

a All values are based on 90 samples representing populations from southwestern China (*N* = 18–27 for each); see Appendix 1 for locality and voucher information.

**Table 2:** Genetic variation in the 11 polymorphic EST‐SSR markers in four *Camellia reticulata* populations.^a^

Locus	TC (*n* = 18)				XD (*n* = 26)				SM (*n* = 19)				CX (*n* = 27)				Total (*n* = 90)		
	*A*	*H*_o_	*H*_e_	HWE	*A*	*H*_o_	*H*_e_	HWE	*A*	*H*_o_	*H*_e_	HWE	*A*	*H*_o_	*H*_e_	HWE	*A*	*H*_o_	*H*_e_
CSSR2	3	0.222	0.541	0.032	3	0.462	0.503	0.237	2	0.000	0.102	0.000^b^	2	0.000	0.140	0.000^b^	3	0.178	0.372
CSSR5	2	0.167	0.322	0.029	3	0.462	0.585	0.000^b^	3	0.053	0.363	0.000^b^	3	0.296	0.319	0.008	3	0.267	0.428
CSSR11	4	0.222	0.630	0.000^b^	4	0.115	0.664	0.000^b^	5	0.000	0.677	0.000^b^	4	0.037	0.505	0.000^b^	5	0.089	0.636
CSSR17	4	0.722	0.589	0.618	5	0.846	0.686	0.000^b^	3	0.053	0.317	0.000^b^	4	0.111	0.357	0.000^b^	7	0.433	0.518
CSSR18	3	0.722	0.643	0.026	5	0.846	0.787	0.000^b^	4	0.421	0.563	0.000^b^	3	0.296	0.377	0.144	6	0.567	0.642
CSSR19	2	0.000	0.508	0.000^b^	2	0.462	0.498	0.705	2	0.000	0.102	0.000^b^	2	0.148	0.201	0.136	2	0.178	0.422
CSSR35	2	0.056	0.056	1.000	3	0.039	0.112	0.000^b^	2	0.000	0.102	0.000^b^	3	0.148	0.322	0.000^b^	3	0.067	0.166
CSSR36	3	0.111	0.641	0.000^b^	4	0.039	0.612	0.000^b^	3	0.000	0.199	0.000^b^	3	0.037	0.238	0.000^b^	4	0.044	0.471
CSSR38	3	0.222	0.298	0.000^b^	4	0.192	0.250	0.000^b^	2	0.000	0.398	0.000^b^	5	0.111	0.357	0.000^b^	5	0.133	0.330
CSSR45	3	0.778	0.560	0.267	4	0.769	0.719	0.012	4	0.158	0.289	0.000^b^	3	0.444	0.444	0.000^b^	5	0.544	0.580
CSSR48	3	0.722	0.532	0.220	2	0.000	0.462	0.000^b^	2	0.000	0.102	0.000^b^	2	0.074	0.352	0.000^b^	3	0.167	0.465
Mean	2.909	0.359	0.484		3.546	0.385	0.534		2.909	0.062	0.292		3.091	0.155	0.328		4.182	0.242	0.457

Note

*A* = number of alleles sampled; *H*_e_ = expected heterozygosity; *H*_o_ = observed heterozygosity; HWE = Hardy–Weinberg equilibrium; *n* = number of individuals sampled.

a Locality and voucher information are provided in Appendix 1.

b Chi‐square test for Hardy–Weinberg equilibrium. Locus showed significant deviations from Hardy–Weinberg equilibrium (*P* < 0.001).

Among the 11 polymorphic loci, the number of alleles per locus ranged from two to seven with a mean of 4.182. The levels of observed and expected heterozygosity were 0.044–0.567 and 0.166–0.642, with averages of 0.242 and 0.457, respectively (Table 2). Four SSR markers were able to detect levels of expected heterozygosity above 0.5, indicating a high level of polymorphism in *C. reticulata*. All 11 polymorphic loci showed deviation from Hardy–Weinberg equilibrium within two or more populations (Table 2) as a result of heterozygosity deficits. This was most likely the result of the reproduction mode, habitat fragmentation, and inbreeding. We found no consistent deviation from linkage disequilibrium for any loci within the populations (*P* < 0.001). Cross‐species amplification of the 11 polymorphic loci was tested on *C. saluenensis*,* C. pitardii*, and *C. yunnanensis*. All 11 EST‐SSR markers were amplified successfully, using the same PCR protocol for *C. reticulata*, and were shown to be polymorphic (Table 3).

**Table 3:** Cross‐amplification and genetic diversity statistics of EST‐SSR markers developed for *Camellia reticulata* in three related species.^a^

Locus	*Camellia saluenensis*				*Camellia pitardii*				*Camellia yunnanensis*			
	JCPT (*n* = 13)				JCPL (*n* = 15)				JCYN (*n* = 12)			
	*A*	*A*_e_	*H*_o_	*H*_e_	*A*	*A*_e_	*H*_o_	*H*_e_	*A*	*A*_e_	*H*_o_	*H*_e_
CSSR2	3	1.476	0.154	0.335	2	1.385	0.067	0.287	2	1.180	0.000	0.159
CSSR5	2	1.257	0.077	0.212	2	1.220	0.067	0.186	1	1.000	0.000	0.000
CSSR11	3	1.660	0.154	0.394	3	1.312	0.067	0.246	3	1.405	0.083	0.301
CSSR17	3	1.733	0.077	0.440	3	1.495	0.133	0.343	2	1.280	0.083	0.228
CSSR18	2	1.451	0.077	0.323	2	1.220	0.067	0.186	2	1.280	0.083	0.228
CSSR19	2	1.451	0.077	0.323	2	1.220	0.067	0.186	1	1.000	0.000	0.000
CSSR35	3	1.751	0.154	0.446	3	1.402	0.133	0.297	3	1.412	0.000	0.304
CSSR36	3	1.808	0.231	0.465	3	1.495	0.067	0.343	3	1.412	0.000	0.304
CSSR38	2	1.257	0.077	0.212	2	1.220	0.067	0.186	2	1.180	0.000	0.159
CSSR45	3	1.660	0.000	0.394	3	1.226	0.067	0.191	2	1.180	0.000	0.159
CSSR48	2	1.257	0.077	0.212	2	1.142	0.000	0.129	1	1.000	0.000	0.000
Mean	2.546	1.514	0.105	0.342	2.455	1.303	0.073	0.235	2.000	1.212	0.023	0.168

Note

*A* = number of alleles sampled; *A*_e_ = effective number of alleles; *H*_e_ = expected heterozygosity; *H*_o_ = observed heterozygosity; *n* = number of individuals sampled.

a Locality and voucher information are provided in Appendix 1.

## CONCLUSIONS

The EST‐SSR polymorphic markers developed in this study will add to the existing resources of molecular markers and are expected to be useful for studies on population structure and genetic diversity in *C. reticulata*. The microsatellite loci described here were successfully cross‐amplified in *C. saluenensis*,* C. pitardii*, and *C. yunnanensis*, suggesting that these markers may also be applicable to the study of genetic diversity in other *Camellia* species.

## Data Availability

Expressed sequence tag sequences for the newly developed primers have been deposited to the National Center for Biotechnology Information (NCBI)'s GenBank database; accession numbers are listed in Table 1 and Appendix 2.

## APPENDIX 1

**Table:** Locality and voucher information for *Camellia* species used in this study.^a^

Species	Population code	Voucher no.	Location	Geographic coordinates	Elevation (m)	*N*
*C. reticulata* Lindl.	TC	CR‐TY‐021	Mazhan, Tengchong, Yunnan, China	25°12′03.65″N, 98°28′34.46″E	1980	18
*C. reticulata*	XD	CR‐TY‐013	Niujie, Xundian, Yunnan, China	25°52′57.8″N, 102°59′14.4″E	2398	26
*C. reticulata*	SM	CR‐TY‐004	Baiyi, Songming, Yunnan, China	25°19′21.03″N, 102°53′28.21″E	2121	19
*C. reticulata*	CX	CR‐TY‐018	Zixi Mountain, Chuxiong, Yunnan, China	24°59′54.33″N, 101°25′10.77″E	2318	27
*C. saluenensis* Stapf ex Bean	JCPT	CS‐TY‐01	Fuyuan, Songming, Yunnan, China	25°15′53″N, 102°55′18″E	2147	13
*C. pitardii* Cohen‐Stuart	JCPL	CP‐TY‐01	Junzi Mountain, Shizong, Yunnan, China	24°37′13.58″N, 104°9′28.4″E	2031	15
*C. yunnanensis* (Pit. ex Diels) Cohen‐Stuart	JCYN	CY‐TY‐01	Machang, Heqing, Yunnan, China	26°28′26.68″N, 100°3′13.53″E	3113	12

Note

*N* = number of individuals sampled.

^a^ All voucher specimens are deposited in the Herbarium of the Kunming Institute of Botany (KUN), Kunming, Yunnan Province, China.

## APPENDIX 2

**Table:** Characteristics of 39 monomorphic microsatellite loci developed in *Camellia reticulata*.

Locus	Primer sequences (5′–3′)	Repeat motif	Expected allele size (bp)	GenBank accession no.
CSSR1	F: CAAAGCCAAATGGAATTGTC	(A)_30_	179	FS943489.1
	R: GCCAGTGAATTGTAATACGA			
CSSR3	F: TTCCTCCATTTGCGTGAAA	(AG)_13_	194	FS951626.1
	R: ACCGTCTAGCCTCCAATC			
CSSR4	F: TCGTCAATTCCTTCTTGTTG	(CTT)_9_	128	FS951901.1
	R: TTGGTTACAGATGGAGATGG			
CSSR6	F: TGTTCTCAATCCACTCTTCA	(TCA)_9_	140	FS953739.1
	R: GCGACAATAATAGGCTCTTG			
CSSR7	F: AAGATGAAAGTGTGGATTCC	(TG)_25_	148	GH159087.1
	R: GTAACAACCATCACCAACAT			
CSSR8	F: GCAGTAGTTGTTGAAGTTGAG	(A)_31_	180	GW863559.1
	R: CCAGTGAATTGTAATACGACTC			
CSSR9	F: TTGTATGTTCCAAGGCATTG	(A)_30_	201	GW863563.1
	R: GACTCACTATAGGGCGAATT			
CSSR10	F: TGCTGTCAACTACCCTTC	(AG)_20_	104	GW342632.1
	R: GGTGCTTGAGTCTGTGAT			
CSSR12	F: ACCTTGGCTTTGCTCTCT	(AAG)_13_	135	GO255031.1
	R: TTGACGCCGAAGACTCTC			
CSSR13	F: TGCTTGCTATCATACAGTTC	(A)_30_	192	GW315083.1
	R: GCCAGTGAATTGTAATACGA			
CSSR14	F: GGATGTGTGTTTAGGACCAT	(A)_30_	196	GW863601.1
	R: ACGGCCAGTGAATTGTAAT			
CSSR15	F: TCTAATGCCAAGCCTCAAC	(A)_30_	179	GH734011.1
	R: GACTCACTATAGGGCGAATT			
CSSR16	F: TCACTAGACCATGTGCTTA	(A)_30_	187	GH734178.1
	R: GTGAATTGTAATACGACTCC			
CSSR20	F: GCAGCTCTCTACTTGTCAT	(A)_31_	206	GH709471.1
	R: GCCAGTGAATTGTAATACGA			
CSSR21	F: GTTGCTAAATCTGTTGCTAC	(A)_30_	177	GH709922.1
	R: GCCAGTGAATTGTAAATACGA			
CSSR22	F: GAACAATGATGACATCTCCA	(CTCCAG)_5_	107	GH709760.1
	R: ATAAGGGAGGAGTGATTTGG			
CSSR23	F: TTGGACACCTTGAATGACT	(A)_28_	114	GH612882.1
	R: TAGTGATTAGCGTGGTCG			
CSSR24	F: TGTATGATAGCAAGCTGAAG	(A)_28_	110	GH613058.1
	R: TAGTGATTAGCGTGGTCG			
CSSR25	F: GCAGCGAGAACTTTGATG	(A)_30_	210	GE650217.1
	R: GCCAGTGAATTGTAATACGA			
CSSR26	F: TCAGACTGTACTTAGTGGTT	(A)_30_	126	GH623471.1
	R: TAGTGATTAGCGTGGTCG			
CSSR27	F: CAGTGGATGATTGGTAATTTGG	(A)_31_	176	GW696815.1
	R: AGTGGTATCAGGGCAGAG			
CSSR28	F: CACATCTCTCCTGTTGCTA	(A)_47_	148	FS944961.1
	R: CTTCTTGCTTGTCTTTCTTC			
CSSR29	F: GCTGTCTGCTTTGTACGA	(GA)_11_	163	FE861335.1
	R: TCTCTTCTCTTCCTCCTCTC			
CSSR30	F: AGAAAGAAGCTGCAAGGG	(TTCT)_5_	128	FE861638.1
	R: CGTAGATGAGGCTGGAAG			
CSSR31	F: ACGCTGAAGTCCAAATCC	(GCC)_6_	145	CV699742.1
	R: AGTGGTCTCCTGTGCTAC			
CSSR32	F: ACACTCACTCAATCACTGTT	(A)_29_	207	GH733834.1
	R: TGTAATACGACTCACCATAGG			
CSSR33	F: GCAAATGTTGGGCTTGTT	(A)_29_	172	FS959890.1
	R: GCCAGTGAATTGTAATACGA			
CSSR34	F: CATCGCATCGTCGTCATC	(TC)_15_	128	FS947495.1
	R: GATCCGACACTTGAACTTGA			
CSSR37	F: ACCCAAAGCAAAGCCAAT	(AG)_13_	103	FS948821.1
	R: AACTAGCTGAAGATAGAGGAG			
CSSR39	F: TTCATCACAGACACCATCA	(TTTC)_8_	100	FS949741.1
	R: TCACCAATCACAAATCACAG			
CSSR40	F: GGCTATGTACTTGATGTTCTTC	(A)_23_	197	FS954738.1
	R: CCAGTGAATTGTAATACGACTC			
CSSR41	F: CCTCCTCTATCTTCGATCAATA	(GA)_10_	110	FS949096.1
	R: CGTTAAAGCCATTTCTCTCT			
CSSR42	F: GTAACGATTGAATCTGGCAT	(A)_20_	186	GH623925.1
	R: TCACTATAGGGCGAATTGG			
CSSR43	F: CGCTATTTATCCTTGCTGTT	(A)_23_	133	GH623383.1
	R: GTGGTATCAACGCAGAGTA			
CSSR44	F: CCACCATCACATCCTTACA	(CAC)_5_	157	FS949501.1
	R: GTGGAGGAGGAGATGAGTA			
CSSR46	F: GCCGTGAAGATAATGTTGG	(A)_64_	149	GH623235.1
	R: TAGTGATTAGCGTGGTCG			
CSSR47	F: CTAGTGATTAGCGTGGTC	(T)_31_(T)_34_	148	GH623319.1
	R: GTTGTGATTACGATCTCTGA			
CSSR49	F: TAACCCTATGTAGACCTCAGT	(A)_31_	215	GW863554.1
	R: CCAGTGAATTGTAATACGACTC			
CSSR50	F: TCCATAAAGGAACCTCTAGC	(CT)_13_	164	JK511141.1
	R: TCCAAACATACTCCCAAACT			
